# Designing AI-generated antimicrobials for targeting bacterial microdomains

**DOI:** 10.1038/s41598-025-31350-1

**Published:** 2025-12-09

**Authors:** Mateusz Rzycki, Adam Gruda

**Affiliations:** Department of Biomedical Engineering, Wroclaw University of Science and Technology, 50-370 Wroclaw, Poland

**Keywords:** Microdomains, Lipid membranes, Diptool, Free energy, Antimicrobials, Drug design, ReLeaSE, Cheminformatics, Computational biology and bioinformatics, Computational models

## Abstract

Bacterial membranes are essential for processes such as nutrient transport, signal transduction, and maintaining cellular integrity. Within these membranes, specialized microdomains like cardiolipin-rich domains, serve as key sites for protein localization, membrane curvature regulation, and stress response. These microdomains possess high anionic headgroup density and specific lipid composition, which can enhance electrostatic and hydrophobic interactions with antimicrobial agents. In this study, we investigated whether antimicrobial compounds can selectively target membranes with distinct lateral lipid distributions. To this end, we modeled two bacterial membrane systems: one with a randomized lipid composition and another featuring an idealized cardiolipin-rich microdomain. Using a generative neural network framework guided by specific molecular design criteria, we generated AI-driven antimicrobial candidates and assessed their membrane interactions via free-energy calculations. Our analysis revealed preferential binding of compounds to cardiolipin-rich domains, evidenced by lower binding energies, alongside higher translocation barriers in these regions, attributable to strong electrostatic anchoring and tight lipid packing. Further clustering and feature importance analysis identified recurring structural motifs associated with potent antimicrobial activity, supporting that cardiolipin-rich regions may facilitate selective binding. Parallel toxicity predictions indicated several top candidates have low predicted toxicity, a critical factor for drug development. Notably, the top AI-designed compounds were benchmarked against established membrane-targeting drugs to assess similarities in their antimicrobial activity profiles. By integrating generative AI with advanced membrane modeling, this work establishes a robust framework for the rational design of new membrane-targeting antimicrobials and highlights how membrane composition influences drug-membrane interactions and efficacy.

## Introduction

Bacterial membranes function as dynamic, heterogeneous structures that not only protect the cell but also facilitate essential processes such as transport of nutrients, signal transduction, and regulation of internal homeostasis^1^. Moreover, these membranes are highly organized, comprising diverse lipid species that can self-segregate into discrete microdomains, each characterized by unique lipid and protein compositions and specialized functions^2^. One class of these microdomains is enriched in cardiolipin (CL), a dimeric phospholipid known for its cone-shaped structure^3^. Cardiolipin-rich regions are involved in critical functions, including protein localization, stress response, and modulation of membrane curvature^4,5^. Studies of CL’s effect on membrane mechanics have concluded with inconsistent results. Several reports indicate that CL increases membrane stiffness and lipid packing compared with other lipids^6–10^, whereas other work suggests that CL-rich regions exhibit greater fluidity^11–13^. Consequently, CL-rich microdomains can differ locally from the surrounding membrane in dynamics and mechanical properties; however, the exact direction of change is still vague. Changes in lipid composition can influence the membrane’s physicochemical properties, including bilayer thickness, fluidity, lateral pressure, and transport behavior^14,15^. Thus, lateral heterogeneity may have profound implications for the activity of antimicrobial compounds, as recent studies suggest that membrane rigidity influences the binding and insertion of antimicrobial molecules^16,17^.

Despite the growing recognition of membrane microdomains as structural and functional hotspots, their role in antimicrobial drug design remains underexplored. The search for effective antibacterial agents has become increasingly urgent in recent years, driven by the escalating crisis of antibiotic resistance among clinically important pathogens^18^. Nevertheless, discovering and optimizing novel chemical entities for antibacterial efficacy remains a formidable challenge. Traditional computational approaches for evaluating membrane-active compounds, such as molecular dynamics (MD) simulations and molecular docking, present notable limitations. Free energy calculations via MD, while accurate, are computationally demanding for large-scale compound screening due to their reliance on extensive sampling timescales. Docking methods, on the other hand, typically focus on protein targets and can fail to capture the complexity of membrane interactions by treating lipids as static and homogeneous environments^19^, thus neglecting the dynamic heterogeneity of microdomains. These may hinder the efficient discovery of compounds that selectively target bacterial membranes, a critical need in the face of increasing antibiotic resistance.

To address these challenges, specialized tools such as Diptool^20^ have emerged as promising alternatives. Diptool leverages coarse-grained models and implicit membrane representation to rapidly estimate the free energy of compound-membrane interactions. Using an optimized force field and streamlined algorithms for calculating membrane insertion energies, Diptool can provide insights into how a compound governs its propensity to bind and penetrate lipid bilayers^21^. Because the bilayer is treated as a fixed array of oriented dipoles and the solvent as a continuum dielectric, Diptool does not include lateral lipid diffusion, explicit water or ions, nor membrane undulations. These approximations preclude the simulation of collective or hydration-dependent phenomena (e.g., pore nucleation or flip-flop) but render Diptool several orders of magnitude faster than fully atomistic MD. This positions Diptool as a well-suited platform for probing hypotheses about how membrane mechanics may influence antimicrobial activity, a paradigm proposed that differences in bacterial and eukaryotic membrane stiffness could underpin the selectivity of octenidine action^17^.

Building on this premise, we therefore focus on whether antimicrobial compounds preferentially target CL-rich microdomains. These microdomains may function as preferential interaction hotspots^5,22^ facilitating the insertion of amphiphilic molecules into the membrane through electrostatic or van der Waals interactions^23^. If validated, this hypothesis would provide a rationale for designing compounds that target bacterial membrane microdomains—a strategy that could improve both potency and selectivity.

In this study, we applied molecular guidelines for the design of effective antimicrobials to generate AI-driven candidates using a generative neural network framework^24^. These guidelines focused on a target range for molecular weight and partition coefficient (log P), parameters that have been extensively correlated with antimicrobial activity in medicinal chemistry and drug development^21^. Numerous studies have demonstrated that logP is closely associated with lipophilicity and that molecular weight often correlates with alkyl chain length factors directly influencing antimicrobial efficacy^25–28^. The resulting compounds were subsequently evaluated via Diptool-driven free-energy calculations to systematically assess their interactions with bacterial membrane models. Two systems were investigated: one featuring a homogeneous membrane with a randomized lipid distribution and another incorporating an idealized CL-rich microdomain. By comparing the behavior of the same compounds across these different environments, we aimed to determine whether microdomain formation serves as a facilitator of drug–membrane interactions. Finally, we grouped compounds by their computed free-energy profiles to pinpoint the most potent antimicrobial candidates. From these clusters, two lead molecules were selected based on selected physicochemical descriptors and energetic signatures. To contextualize our novel designs, we likewise parameterized and evaluated a panel of well-characterized membrane-targeting drugs. Toxicity—critical in drug discovery—was also evaluated using ProTox 3.0^29^, and a feature importance analysis was performed to reveal the principal physicochemical properties essential for the design of efficient antimicrobials.

Our study integrates three innovative research areas: the influence of membrane composition on antimicrobial efficacy, the use of generative AI in drug design, and the application of advanced computational methods for modeling membrane interactions. By combining these approaches, we aim to enhance the rational design of antimicrobials targeting pathogenic membranes.

## Methods

### AI-driven agents

To generate molecules with the desired characteristics, we employed a modified Reinforcement Learning for Structural Evolution (ReLeaSE) framework^24^. ReLeaSE integrates two neural networks—a generative model and a predictive model—that are trained separately and then used jointly. The generative network is a stack-augmented recurrent neural network (RNN), which leverages an external memory stack to learn the grammar of SMILES strings better. This architecture enables the generation of chemically valid and diverse molecules by capturing long-range dependencies in the SMILES representation (e.g., matching rings and brackets). The predictive network is a property predictor trained to estimate key molecular properties (here logP and molecular mass) for any given structure. A detailed description of the ReLeaSE network was delivered elsewhere^24^.

In practice, we first used the ReLeaSE architecture and pre-trained the generative RNN on a large chemical library to learn general chemical syntax. Then trained the predictive model on compounds with known logP and molecular weight values (to establish a quantitative structure–property relationship). In our case, the predictive model was tuned, encouraging the production of molecules that satisfy our design criteria (masses 490–610 g/mol and logP 8–11). These guidelines are based on two complementary studies of gemini surfactants-dimeric amphiphiles whose antimicrobial activity has been experimentally validated. Briefly, we assembled a database of 250 agents and evaluated each using our molecular dynamics pipeline^30^. Comparative *in* *silico* analysis showed that the compounds exhibiting the greatest membrane-disruption potency clustered tightly within defined molecular-weight and lipophilicity windows. From these observations, we derived design rules for next-generation antimicrobial agents^21^. We also monitored the diversity of the candidates generated by tracking the structural similarity fraction with the Tanimoto coefficient (mean *J = 0.33*).

**Fig. 1:**
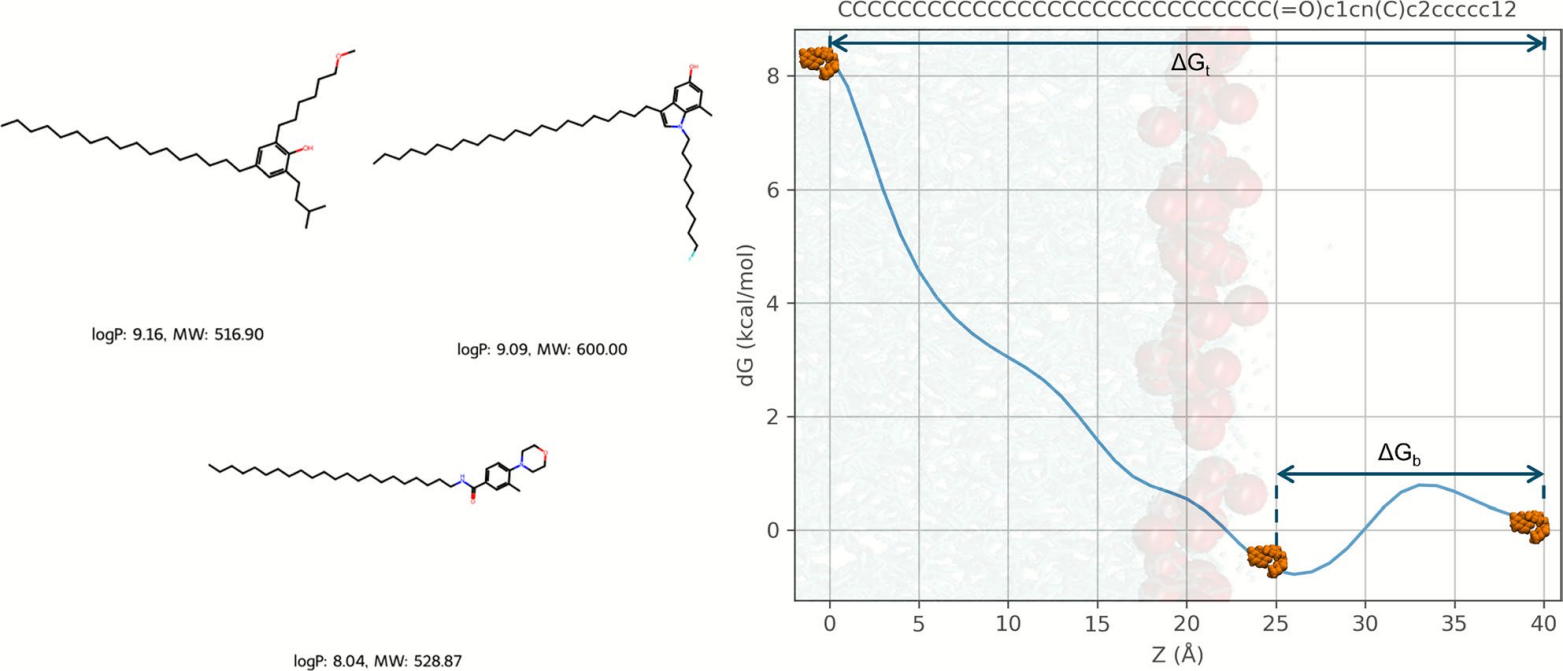
(Left) Exemplary AI-driven agents. (Right) Diptool free-energy profile of compound–membrane interactions, overlaid on a membrane background for clarity (headgroups colored in red, interior in blue; agent in orange). The profile is constructed along the Z-axis, with the initial drug position ($Z=40$Å) and membrane center ($Z=0$Å). The binding energy $\Delta G_{b}$ denotes the minimum at the membrane surface, and the translocation energy $\Delta G_{t}$ denotes the barrier to reach the membrane core.

After thorough structural validation, 73 molecules were deemed suitable for further investigation. Exemplary structures of AI-driven agents are presented in Figs. 1 and S1. Distributions of key physicochemical properties (log P and molecular weight) and SMILES string lengths across the entire library are shown in Figs. S2 and S3, respectively. All AI-generated compounds in this study were drawn from the ChEMBL database under the constraint of neutral net charge, and subsequent verification confirmed a formal charge of zero for each molecule. A complete summary of these compounds, including SMILES, calculated dipole moments, and estimated free energies, is provided on GitHub and a supplementary spreadsheet (.csv).

### Agent parametrization

**Fig. 2:**
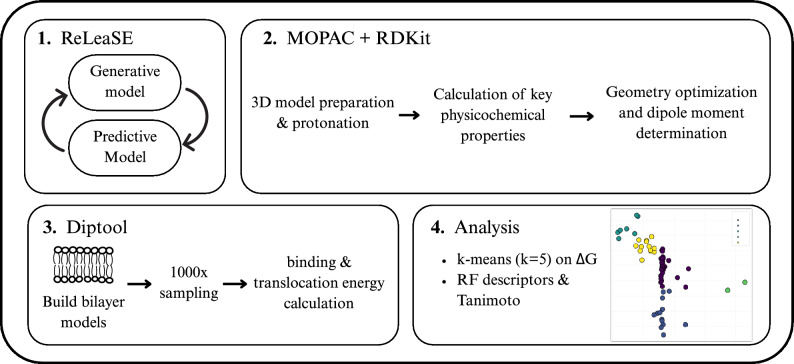
Four-step computational pipeline: (**1**) generative + predictive modelling (ReLeaSE); (**2**) quantum/cheminformatic property calculation (MOPAC + RDKit); (**3**) membrane binding and translocation energies (Diptool, 1000 samples); (**4**) *k*-means clustering ($k = 5$) on $\Delta G_{\text {trans}}$ and $\Delta G_{\text {bind}}$ values, followed by RF-ranked RDKit descriptors and Tanimoto similarity to select leads.

Figure 2 presents the end-to-end computational workflow developed in this study. The generated molecules were parameterized following the established Diptool framework^21^. Initially, the SMILES strings of the compounds were converted into three-dimensional molecular structures using the RDKit cheminformatics library in Python^31^. Hydrogen atoms were added to ensure proper protonation states. Molecular descriptors, including molecular weight and logP, were also calculated with RDKit to characterize the physicochemical properties of the compounds. Subsequently, the dipole moments of the compounds were determined from their geometry-optimized low-energy conformations. These calculations were performed using the MOPAC software package (Molecular Orbital PACkage)^32,33^, using the semi-empirical PM7 method with implicit solvation to account for aqueous conditions. The effects of the solvent were modeled using a dielectric constant (EPS = 78.5), and all calculations were carried out under the assumption of a neutral net charge.

### System preparation

Membrane simulations were performed using Diptool, which enables rapid estimation of free energy and evaluation of antibacterial potential on model membranes. Two distinct membrane construction approaches were employed to assess the affinity of compounds on microdomains. In the first approach, lipids were randomly distributed across both bilayer leaflets. The second approach involved modeling a circular, idealized microdomain surrounded by other lipids, simulating phase separation as observed in bacteria such as *Escherichia* *coli*^4^, in *Bacillus* *subtilis*^16^ and *Staphylococcus* *aureus*^34,35^. In Diptool, the membranes were represented as sets of dipoles that interact with each other as well as with the agent dipoles. Dipole moments for individual lipids were obtained from 200 ns MD simulations (see Table S1). The detailed MD protocol has been described elsewhere^17^. Lipid parametrization followed the established Diptool methodology^20^, and the area per lipid was estimated using Voronoi diagrams.

Both bacterial membrane models comprised 722 lipids, symmetrically distributed in a ratio of 0.4 cardiolipin (CL), 0.4 phosphatidylethanolamine (PE), and 0.2 phosphatidylglycerol (PG). This composition was chosen to emulate the local enrichment of CL observed at the poles and septa in E. coli or B. subtilis, rather than the global lipidome^36,37^. Although it varies by species, this mixture provides a neutral-charged, PE-abundant background in which CL microdomain behavior can be investigated, maintaining the total negative charge of the membrane. Active compounds were initially positioned 40Å above the membrane center in an implicit water environment characterized by a dielectric constant and viscosity^20^.

Given the stochastic nature of Diptool simulations, 1000 iterations were performed for each molecule to ensure robust sampling of the free energy landscape. This extensive sampling strategy was implemented to account for variability in compound-membrane interactions and to enhance the statistical reliability of the results. Free energy profiles were derived from these simulations, with a total of 146 000 runs conducted across all 73 AI-generated molecules, yielding 146 energy profiles. The resulting data were analyzed employing a histogram-based approach with a bin width of 1Å along the membrane normal, ensuring appropriate resolution of the energy minima and barriers associated with the compound insertion. As stated, Diptool derives dipole moments for the geometry corresponding to the lowest energy orientation. To account for orientation variability, 1000 independent simulations were performed with slight perturbations to the dipole moments, sampling alternative insertion angles. Only those trajectories that reach the membrane center, i.e., well-oriented insertion events, contribute to the free-energy profile. This approach captures the energetics of favorable orientations while excluding poorly oriented paths^20,21^. An example Diptool free energy profile is shown in Fig. 1, where the x-axis refers to the reaction coordinate (perpendicular to the membrane surface). Here, two key properties were highlighted: the binding energy ($\Delta G_{b}$) and the translocation energy ($\Delta G_{t}$). The binding energy is defined as the lowest free energy observed near the membrane surface ($\pm 5$Å), while the translocation energy is calculated as the difference between the free energy at the membrane center. It is estimated based on the distance between the initial position of the center of mass (COM) of the antimicrobial agent (modeled as a sphere with defined size, orientation, and partition coefficient) and the geometric center of the lipid bilayer along $Z-axis$, i.e.,

$$\begin{aligned} \Delta G_{t} = \Delta G_{(Z=0)} - \Delta G_{(Z=40)}. \end{aligned}$$

Similarly, $\Delta G_{b}$ is determined as the difference between the free energy at the binding site and the free energy at the initial state.

### Clustering methods and molecular descriptors

To identify the most promising groups of compounds that interact with both microdomain-rich membranes and randomized lipid arrangements, we employed k-means clustering. This method groups compounds into categories based on similarities in selected features, making it suitable for uncovering patterns within multidimensional data sets. Specifically, we clustered the molecules according to two key energy metrics: the lowest transition energies (reflecting the energy barrier for membrane insertion) and the lowest surface interaction energies (indicating the affinity for membrane binding). The analysis was performed using five clusters, determined by the elbow graph method and implemented via the scikit-learn library in Python^38^.

Molecular descriptors are essential for understanding the physicochemical and biological properties of chemical compounds, as they provide quantitative representations of molecular structures and facilitate the prediction and classification of various properties. In this study, we used Morgan fingerprints (2048-bit binary vectors representing substructural features) generated for all molecules, and subsequently used these to derive 208 chemical descriptors via the RDKit package^39^. Each descriptor provides specific information about molecular features, such as size, polarity, and functional group composition.

To identify the most informative descriptors, we employed a random forest-based feature ranking approach using an 80%/20% train/test split. Model performance on the held‑out test set was strong ($R^2_{score}$ = 0.84; mean absolute error = 0.21; root mean squared error = 0.26). To enhance interpretability, we computed SHapley Additive exPlanations (SHAP) values for each molecular descriptor and ranked all features by their mean absolute SHAP impact. From this ranking, we selected the six descriptors with the greatest influence on model predictions, delivering interpretative analysis.

Furthermore, we evaluated the structural similarity among the compounds by calculating the Tanimoto similarity matrix based on the Morgan fingerprints. The Tanimoto coefficient, which ranges from 0 (no similarity) to 1 (complete similarity), was used to quantitatively assess the degree of structural similarity between the generated molecules to verify chemical diversity or common structural motifs.

## Results and discussion

In this study, we implemented recently published molecular guidelines for designing effective antimicrobials^21^ within a generative neural network framework to obtain AI-driven antimicrobial candidates^24^, which were subsequently tested using Diptool. It allows for quick estimation of the free energy associated with drug–membrane interactions, as well as the energetic barriers imposed by the membrane. Lower free energy values can serve as a promising indicator of high affinity and effective penetration of cell membranes.

**Fig. 3:**
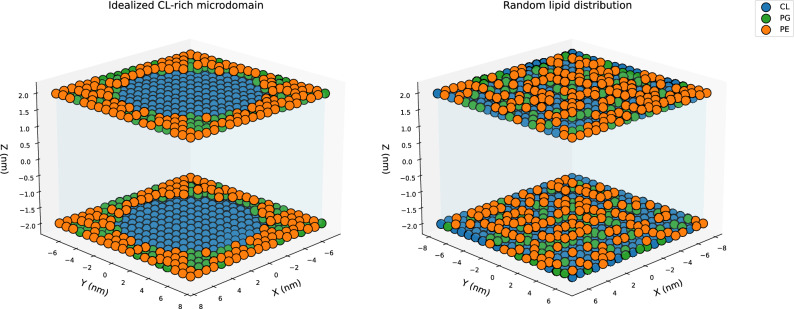
Schematic representation of the two bacterial membrane models analyzed in this study. The left panel represents an idealized cardiolipin-rich microdomain, whereas the right panel features a randomized lipid distribution. Phosphatidylethanolamine (PE) is shown in orange, phosphatidylglycerol (PG) in green, and cardiolipin (CL) in blue.

Our approach is designed to test whether antimicrobial compounds preferentially target CL–rich microdomains, which have been reported to exhibit divergent mechanical behaviors. Several studies describe CL-enriched regions as more rigid and tightly packed^6–8^, whereas others observe increased fluidity within these domains^11–13^. Regardless of these inconsistencies, CL microdomains may serve as preferential interaction hotspots^5,22^. By comparing compound affinity for randomized versus microdomain-enriched bilayers, we directly assess whether CL-rich regions enhance selective antimicrobial binding and penetration. To this end, we modeled two types of membranes with Diptool: one enriched in microdomains and the other with a random lipid distribution (see Fig. 3). Both membrane models share the same lipid composition; however, they differ in lipid arrangement. The central microdomain was constructed using CL, which was surrounded by a mixture of other bacterial lipids (PE and PG).

Having established the physicochemical profile of the AI-designed compounds, we next examined how they interact with model membranes. We computed two free energy values for the tested compounds: the translocation free energy ($\Delta G_{t}$) required for the complete passage of the molecule through the membrane and the binding free energy ($\Delta G_{b}$) associated with reaching the membrane surface. Translocation energy here describes the passage of the compound from the aqueous phase (Z = 40 Å) to the bilayer center (Z = 0 Å) along the membrane normal. In Fig. 1, schematic insets illustrate these positions relative to the headgroup and core regions. It provides an estimate of the compound’s ability to overcome membrane energetic barriers, while the latter evaluates the binding energy between the molecule and the negatively charged hydrophilic lipid head groups. By quantifying both $\Delta G_{t}$ and $\Delta G_{b}$, we capture two complementary antimicrobial modes of action. A low $\Delta G_{t}$ typically correlates with deep membrane insertion and pore formation, whereas a high $\Delta G_{b}$, even when $\Delta G_{t}$ remains elevated, favors surface-bound mechanisms such as carpet-like disruption or toroidal-pore induction at the lipid headgroup interface. Both energy values are critical when designing membrane-active antimicrobials, whose modes of action span a continuum from transient pore formation or detergent-like solubilisation to subtler effects such as altering lipid packing, modulating microdomain organization, inducing curvature stress or dissipating membrane potential. For example, the lipopeptide daptomycin^16^ and the $\beta$-peptidomimetic AMC-109^34^ kill bacteria chiefly by condensing anionic microdomains and mis-localising cell-wall synthesis enzymes rather than perforating the bilayer, a paradigm that reinforces the need to interpret our $\Delta G_{b}/\Delta G_{t}$ landscape within this broader mechanistic spectrum^40^.

**Fig. 4:**
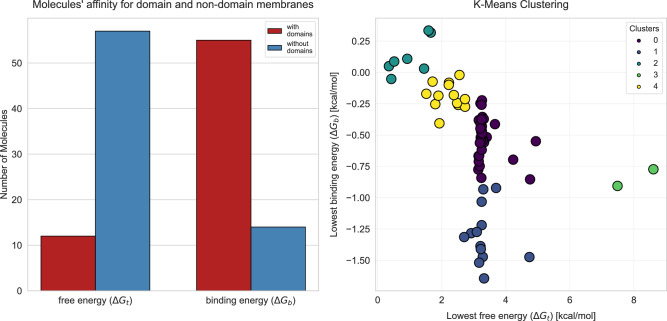
(Left panel) The distribution of compounds with lower translocation and binding energy values on membranes with randomized lipid distribution and those with microdomains. (Right panel) K-means clustering based on minimal translocation and binding energies, with clusters 1 and 2 representing the most promising candidates due to their consistently low energy values.

The left panel of Fig. 4 shows the number of compounds for which we observed lower free energy values during translocation and surface binding. Notably, for 61 compounds, lower $\Delta G_{t}$ were recorded in membranes with a randomized lipid distribution, while only 12 compounds exhibited lower $\Delta G_{t}$ in microdomain-enriched membranes. Conversely, when examining the binding energy $\Delta G_{b}$, 59 compounds showed lower energies in the microdomain-containing membrane. These findings suggest that microdomains may facilitate the effective binding of compounds at the membrane surface, while simultaneously hindering further membrane penetration due to the high energetic barrier. This phenomenon may be directly attributed to the high local charge density generated by the anionic nature of cardiolipin head groups, which attract compounds via electrostatic interactions and preferential van der Waals contacts^22^. Moreover, London dispersion interactions between the molecular moieties further contribute to the stabilization of membrane association. It is hypothesized that increasing the net positive charge may strengthen electrostatic interactions with negatively charged microbial membranes, thereby enhancing antimicrobial activity^41^. This could facilitate the formation of pores or channels within the membrane, or promote membrane solubilization into micellar structures^42^. These attractive forces may arise from the distribution of dipole moments within AI-designed agents. It is well established that molecular charge is closely related to dipole moment, and the presence of charge significantly influences molecular reactivity and toxicity, particularly through adsorption processes^43^. Charged molecules are generally more readily adsorbed onto surfaces than their uncharged counterparts. Although the precise relationship between dipole moment and molecular reactivity remains incompletely understood, numerous studies have reported a general trend of increased reactivity with higher dipole moments^44^. In a random lipid distribution system, no clear correlation was observed between $\Delta G_t$ and total dipole moment. However, in systems with CL-rich domains, a strong correlation was evident ($R^2 = 0.97$), supporting the proposed relationship. It should also be noted that $\Delta G_b$ exhibited a negative correlation with the total dipole moment.

Thus, even formally neutral compounds may exploit intramolecular forces to achieve selective targeting of negatively charged bacterial domains. Consequently, the overall approach of compounds towards microdomains is preferential, yet the lateral organization or high re dipole moment may impede further penetration.

Based on these energy profiles, we performed k-means clustering to identify patterns among the compounds. Following the interaction energies of the agents with different types of membranes, we performed a cluster analysis using both the lowest $\Delta G_{t}$ and the minimal $\Delta G_{b}$ (see Fig. 4). Furthermore, we implemented alternative clustering approaches that consider only the differences in full translocation free energies $\Delta G_{t}$ between the domain and non-domain membranes (see Fig. S4) and only the minimal approach free energies $\Delta G_{b}$ between these two membrane types (see Fig. S5). This approach enabled us to evaluate the compounds’ energy profiles and identify clusters of candidates with the highest affinity for bacterial membranes. In particular, molecules assigned to clusters 1 and 2 attracted our greatest interest.

**Table 1:** Key descriptors for the AI-generated lead candidates and selected reference membrane-active agents.

Molecule ID	Cluster	$\log P$	$M_w$ (Da)	$\Delta G_{\text {t}}^{*}$ (kcal mol$^{-1}$)	$\Delta G_{\text {b}}^{*}$ (kcal mol$^{-1}$)	LD$_{50}$ (mg kg$^{-1}$)	Tox. class (ProTox)	SA score
23	2	9.3	555	1.6	0.3	6430	6	2.9
57	1	8.9	585	3.3	−0.9	10000	6	2.8
68	1	8.3	578	3.2	−1.4	4000	5	2.2
Octenidine	–	9.6	551	15.9	−2.2	800	4	2.9
Chlorhexidine	–	3.3	505	15.1	−2.2	1100	4	2.8
Daptomycin	–	−5.6	1621	35.6	−5.5	2000	5	7.9
Fabimycin	–	2.3	405	14.2	−2.4	500	4	3.8
AMC-109 (LTX-109)	–	3.4	788	15.6	−1.9	840	4	4.4

$^{*}$ - lowest free energy

Our AI-generated candidates exhibit a profile that can be contextualized by comparing them to known membrane-targeting antimicrobials. Notably, the binding affinity patterns we observed (strong surface binding to cardiolipin-rich domains but hindered deep penetration) mirror the behavior of certain existing antibiotics. As a reference framework, Table 1 represents AI-selected leads with five established membrane-active antimicrobials.

Our analysis begins with the octenidine and chlorhexidine, two cationic surfactants that bind avidly to anionic lipid head groups, yet rarely penetrate deeply into the bilayer.. We have previously shown that octenidine’s membrane selectivity correlates more with membrane rigidity than with simple charge attraction, causing it to condense rather than perforate CL patches^17^. This mechanical sensitivity may align with the differential behavior of our candidates across ordered and disordered membrane domains. A similar picture emerges for chlorhexidine; recent MD-NMR work demonstrated that the molecule relocates to the head-group/ester interface, where its cationic termini act as a “molecular staple”, rigidifying the surface without driving deep insertion^45^. In our data set, the cluster 2 leads mirror this behavior (see Fig. 4). They exhibit very low binding energies at the membrane surface, yet face only modest barriers to deeper insertion, suggesting a surfactant-like mode that perturbs local order rather than forming stable pores.

A different strategy is adopted by the cyclic lipopeptide daptomycin and the small $\beta$-peptidomimetic AMC-109 (LTX-109). Fluorescence microscopy and MD simulations show that daptomycin localize to anionic lipid domains and clusters fluid CL / PG nanodomains and mislocalizes late stage cell wall enzymes without fully traversing the bilayer^16,46^. It was found that cardiolipin-rich regions can sequester daptomycin in the outer leaflet, preventing complete membrane permeabilization^46^. This is analogous to our finding that CL-rich microdomains attract our compounds (lower $\Delta G_{b}$ at the surface) but also impose a higher translocation energy barrier ($\Delta G_{t}$).In effect, both our *de novo* molecules and known drugs like daptomycin exploit the high anionic charge of bacterial microdomains for initial binding. Melcrová et al. recently demonstrated that AMC-109 behaves analogously, inserting into the outer leaflet of bacterial membranes while dissolving membrane nanodomains without pore formation^34^. Our cluster 1 compounds reproduce a similar energy landscape: they display a pronounced minimum in the free-energy profile near the headgroup region, but encounter translocation barriers of approximately 10 kcal mol$^{-1}$ as they approach the bilayer center. This combination of strong peripheral anchoring with a substantial energetic hurdle to full penetration is consistent with the domain-clustering and domain-dissolution mechanisms established for daptomycin and AMC-109.

Finally, fabimycin exemplifies a dual‑mode strategy in which a compound exerts rapid surface-driven stress on the bacterial envelope while also targeting intracellular machinery^47^. In our MD study, we demonstrated that fabimycin not only inhibits the FabI enzyme, but also significantly alters membrane mechanics, improving bilayer compressibility and reducing lipid diffusion at the interface^48^.

Furthermore, the behavior of several of our compounds aligns with that of antimicrobial peptides like octapeptins and melittin, after an initial electrostatic docking step these peptides integrate partially into bilayers, cause curvature stress, and transiently permeabilize the membrane. In the case of melittin, this process culminates in the cooperative assembly of toroidal pores, whereas our small molecules appear to create only localized disruptions^49–51^.

Collectively, these findings validate our $\Delta G_{\textrm{b}}/\Delta G_{\textrm{t}}$ screening criteria: the AI-driven agents have recapitulated two established antimicrobial modalities-surface-condensing surfactants and domain-dissolving lipopeptides-while also exploring novel scaffolds within comparable energetic windows, thus offering new chemicals ready for further optimization.

Additionally, we calculated translocation free-energy barriers for several well-characterized membrane-active drugs and compared them with values reported from MD–based potential of mean force (PMF) studies. Diptool predicts barriers of approximately 15.9 and 15.1 $kcal \cdot mol ^{-1}$ for octenidine and chlorhexidine, respectively, which align with the  11 and  15 $kcal \cdot mol ^{-1}$ values reported in MD-PMF analyses^20,52^. Likewise, the lipopeptide daptomycin exhibits a high insertion barrier in both approaches (Diptool: 35.6 $kcal \cdot mol ^{-1}$; MD-PMF:  36 $kcal \cdot mol ^{-1}$), consistent with its propensity to remain sequestered in the outer leaflet of anionic bilayers^53^. For the dual-mode antibiotic fabimycin, our predicted barrier (14.2 $kcal \cdot mol ^{-1}$) closely matches the  13 $kcal \cdot mol ^{-1}$ value from recent simulations^48^. In the case of the peptidomimetic AMC-109, Diptool yields a barrier of  15.6 $kcal \cdot mol ^{-1}$, although no direct PMF data are currently available for comparison. It is important to interpret these numerical comparisons with caution, as the literature PMFs were derived using varied membrane models (typically highly anionic, PG-rich bilayers). Nonetheless, the overall correspondence in barrier heights demonstrates that Diptool can accurately capture membrane-permeation energetics while incurring substantially lower computational cost than conventional MD-PMF methods.

**Fig. 5:**
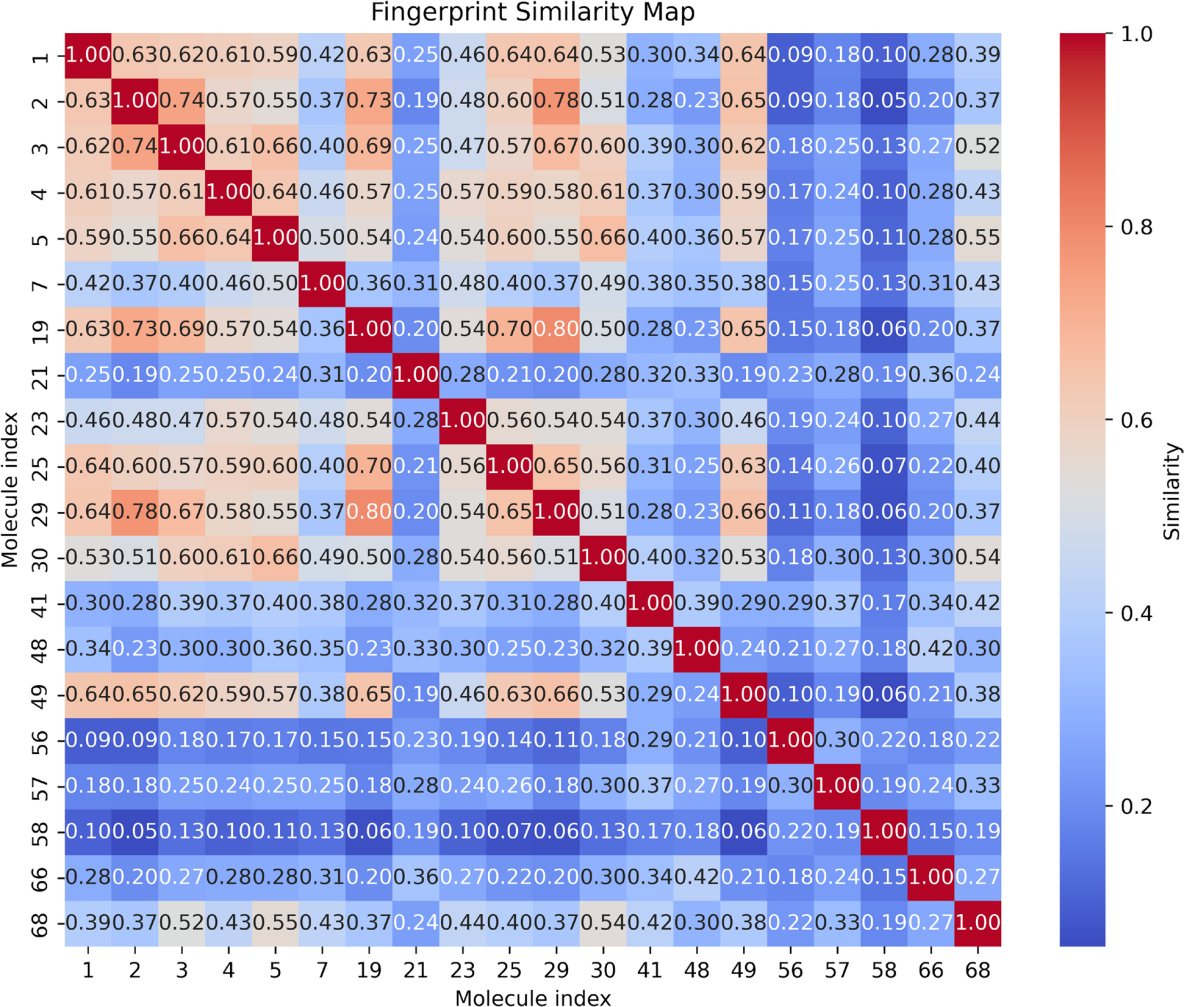
Tanimoto similarity matrix based on molecular fingerprints only for the selected AI-driven agents. The resulting heatmap indicates the structural likeness between two molecules. The similarity ranges from 0 to 1 (no and complete similarity), focusing on clusters (1-2) identified as most relevant based on free energy profiling. The molecular index corresponds directly to the ID provided in the Supplementary Material spreadsheet.

Consequently, we examined the structural characteristics of the 1 and 2 groups in greater detail to assess the degree of molecular similarity and to determine consistent structural parameters that could contribute to effective antimicrobial activity. To this end, we generated 2048-bit Morgan fingerprints for each compound and calculated pairwise Tanimoto coefficients. The resulting similarity matrix is shown as a heatmap in Fig. 5, where each cell indicates the structural likeness between two molecules. Figure 5 illustrates the molecular similarity matrix based on Morgan fingerprints. The highest score (0.8) was observed between compounds 19 and 29, while the lowest (0.05) occurred between compounds 2 and 58. Notably, the first 11 compounds exhibit a greater mutual similarity. This may be attributed to their linear structures and extended hydrophobic chains, often terminating with aromatic groups. The average molecular similarity between the agents is approximately 0.4. However, despite structural diversity, some recurring motifs correlated with potential antibacterial activity were identified.

Specifically, 18 out of 20 compounds contain extended hydrophobic aliphatic chains, which are known to enhance membrane permeability by integrating into bacterial lipid bilayers and disrupting their fluidity and integrity^54^. Additionally, fifteen compounds feature aromatic rings or nitrogen-containing heterocycles, such as pyrimidine and quinoline. These moieties can promote $\pi$-$\pi$ stacking with aromatic residues in bacterial enzymes or intercalating in nucleic acids^55,56^. Seven compounds incorporate nitrogen heterocycles (e.g., piperazine, morpholine), which can promote hydrogen bond formation or involve electrostatic interactions^57,58^. Three compounds contain halogens, and two feature nitro groups. The presence of fluorine atoms, as seen in compounds 41 and 48, has been reported to improve metabolic stability and membrane penetration by increasing lipophilicity and reducing polar surface area^59,60^. Finally, three compounds include sulfur atoms, such as a thiophene ring in compound 21, which may enhance membrane interaction or form disulfide bridges with bacterial proteins, similar to the mechanisms observed with certain sulfonamide antimicrobials^61^.

**Fig. 6:**
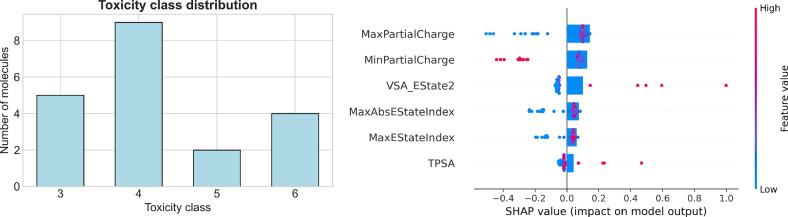
(Left panel) Toxicity data results for the selected compounds. Toxicity classes range from 1 to 6 indicating critical and non-toxicity, respectively. (Right panel) Top six influential descriptors ranked by their importance in predicting free energy interactions using a RandomForest and SHAP analysis. The descriptors capture key electronic, electrostatic, and steric properties that influence compound-membrane binding and penetration.

Given the promising antibacterial activity observed in our compounds, we next evaluated their toxicity profiles to assess their suitability for further development. To this end, we employed ProTox 3.0, a machine-learning platform^29^ that classifies small molecules into six acute toxicity classes, from class 1 (highest toxicity) to class 6 (lowest). As shown in Fig. 6, five compounds fell into class 3, nine into class 4, two into class 5, and four into class 6. Classes 5 and 6 represent lower toxicity risks, indicating that a partial subset of our candidates may offer good antibacterial effects and reduced toxicity, aligning with a broader trend in next-generation antibiotics that combine high efficacy with low host toxicity. A unifying theme among the safer agents is the absence of halogen moieties or aldehyde fractions and an optimized balance of hydrophobicity and polarity. Overly lipophilic molecules tend to cause off-target membrane disruption and toxicity. In fact, highly lipophilic, low-polarity compounds are significantly more likely to exhibit* in vivo* toxicity than those with moderate lipophilicity and sufficient polar surface area^62^. Conversely, compounds falling within an optimal logP range and lacking known toxicophores are associated with reduced toxic effects^63^. Low-toxicity profiles observed among our compounds are reminiscent of certain optimized peptide-based antimicrobials and synthetic derivatives with reduced systemic impact. For instance, modified polymyxin analogs and tryptophan-containing lipopeptides have demonstrated effective membrane disruption with minimized eukaryotic toxicity, attributed to selective insertion into anionic bacterial bilayers and limited interaction with zwitterionic host membranes^34,64^. Our AI-generated leads were designed with physicochemical constraints (e.g. molecular weight and logP limits) aimed at drug effectiveness^21^. These attributes, together with preliminary toxicity predictions, suggest that the AI-designed antimicrobials could achieve high potency against bacteria while maintaining low systemic toxicity, paralleling other modern antimicrobial candidates that have robust efficacy yet favorable safety profiles in development.

Finally, we investigated the significance of various descriptors in influencing the free energy values generated for translocation and binding to the membrane surface. This analysis is particularly relevant in the context of structure-activity relationships, as it allows for the identification of key structural features that modulate antibacterial activity. To this end, we employed a feature engineering approach with a RandomForest model and SHAP analysis to rank descriptors based on their importance. Figure 6 presents the descriptors with the highest impact on the calculated free energy values. SHAP analysis shows that the descriptors related to local charge distribution and polar surface properties have the greatest impact on predicted membrane insertion energetics. Compounds with higher maximum partial charges exhibit positive SHAP contributions impacting the free-energy barrier, while low maximum charges exhibit negative contributions. Extreme negative minima in the partial-charge distribution increase the impact of the barrier, whereas less negative minima promote penetration. A similar trend has been reported for chlorhexidine: rather than its partial charges, an overall increase in the molecule’s net positive charge led to a substantial elevation of the free-energy barrier^52^. Additionally, the *MinPartialCharge* descriptor, which represents the smallest partial charge among all atoms, provides insights into the presence of non-polar regions within the molecule, while the *MaxPartialCharge* descriptor identifies the most strongly charged site. Both descriptors may play a critical role in mediating the hydrogen bonding and electrostatic interactions between the active agents and the negatively charged components of the membrane. Among surface descriptors, larger values of *VSAEState2* and higher *EState* indices both shift predictions toward greater insertion penalties, reflecting the energetic cost of accommodating extensive electrostatic surface regions. *VSAEstate2*, integrates the van der Waals surface area with the E-state indices, reflecting the contribution of atoms with specific electronic properties along with their accessible surface area. This metric can indicate the propensity of a molecule to interact with the hydrophobic regions of bacterial membranes. Furthermore, *MaxEStateIndex*, which encapsulates the overall electronic properties of the molecule, indirectly influences aspects such as molecular dipole and reactivity. Interestingly, descriptors such as *MaxEStateIndex* and *VSAEstate* have previously been identified as significant in other datasets of potential antibacterial agents^21^. The *Topological Polar Surface Area* (TPSA) is defined as the sum of the surface areas of all polar atoms and hydrogen bond formation. TPSA provides information on how well a molecule can penetrate biological membranes. Thus, a higher TPSA value often indicates greater polarity, which can enhance binding but limit a molecule’s ability to pass through the hydrophobic lipid layer. In general, the focus of these descriptors is on the non-covalent forces, such as electrostatic and van der Waals interactions, that drive drug attraction toward bacterial membranes. They summarize key molecular features that include charge distribution, hydrogen-bonding potential, and polar surface area, which determine the strength and specificity of drug–membrane interactions. The role of these interactions is well documented in the literature since several studies attribute the mode of agent action to electrostatic attraction^65^.

**Fig. 7:**
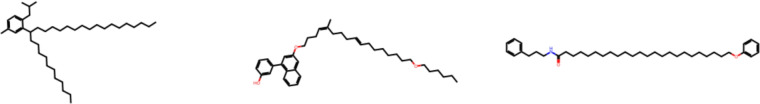
Chemical structures of the three top‑ranked antimicrobial candidates (ID: 23,57,68) selected for experimental validation.

In conclusion of this study, we highlight three specific compounds from the top-ranked groups (clusters 1 and 2) as the most promising antimicrobial candidates for future experimental validation (see Fig. 7). These three molecules were prioritized because they combine optimal predicted binding energies (indicating strong target affinity) with an optimal balance of drug-like properties. In particular, each compound was predicted to have low toxicity and a favorable synthetic accessibility score (SA < 3), suggesting they can be practically synthesized and are less likely to exhibit off-target effects. They also maintain moderate topological polar surface areas (TPSA < 130 Å$^2$) alongside optimal dipole moments (<10 Debye), a combination that implies a balanced amphiphilic character, sufficient polarity to interact with bacterial targets^66,67^. Structurally, both compounds are large, amphiphilic molecules featuring extended aliphatic chains linked to aromatic moieties via stable bonds (amide or ether linkage). This amphiphilic architecture is reminiscent of membrane-targeting antimicrobials, as the polar/aromatic regions engage in binding with biomolecular targets, while the hydrophobic chains could facilitate deeper insertion into bacterial membranes. Notably, neither compound contains potentially problematic functional groups, such as nitro substituents or halogens. The absence of nitro groups (often associated with mutagenicity) and halogen atoms (which can sometimes impart undesired toxicity or metabolic instability) suggests a more favorable safety profile and simpler medicinal chemistry optimization. Notably, ProTox classification placed our top candidates in the lowest toxicity categories, whereas benchmark membrane-active agents such as octenidine and daptomycin fall into higher-toxicity (see Table 1). This shift toward reduced toxicity suggests that our AI‑designed compounds may offer a wider therapeutic window and lower risk of off‑target effects, reinforcing their suitability for further experimental validation. Furthermore, the leading candidates displayed low SA scores (where lower values denote greater ease of synthesis), underscoring their feasibility for chemical preparation (see Table 1). Thus, compounds can be generated both efficiently and at scale, minimizing the time and cost associated with lab work.

Although this study employs a computational approach, we acknowledge that the experimental validation of the top AI-generated candidates is crucial. This validation could be conducted at two levels: using whole *E*.*coli* and model bacterial membranes such as giant unilamellar vesicles (GUVs). In *E*.*coli*, antibacterial efficacy can be assessed through minimum inhibitory concentration assays, employing standard broth microdilution protocols to determine the lowest concentration required to inhibit visible bacterial growth^68^. Membrane integrity can be evaluated through propidium iodide (PI) uptake, reporting inner membrane disruption together with 1-N-phenylnaphthylamine (NPN) fluorescence to quantify outer membrane permeabilization^69,70^. In this context, membrane perturbation would be inferred from rapid increases in PI and NPN fluorescence following exposure to antimicrobial candidates. At the membrane model level, microfluidic trapping of GUVs enables single-vesicle kinetic measurements of membrane disruption, while traditional carboxyfluorescein dye leakage assays provide a high-throughput readout of bilayer permeabilization^71–73^. Furthermore, atomic force microscopy offers a means to visualize compound-induced defects in supported bacterial-mimetic bilayers and to map nanoscale morphological and mechanical alterations in *E*.*coli*^74,75^. When integrated with multiscale high-resolution molecular dynamics simulations^48,76^, this suite of experimental and computational techniques provides a comprehensive framework to highlight how the designed molecules may disrupt bacterial membranes.

In summary, these three candidate agents exemplify the key characteristics identified in our computational screen for potent antimicrobials. Their physicochemical profiles make them suitable candidates for further investigation, and we propose that they be prioritized for synthesis and *in* *vitro* testing to validate their predicted antimicrobial efficacy.

## Conclusions

In summary, our work successfully integrated generative AI with computational membrane modeling to design and evaluate novel antimicrobial agents. Guided by molecular design criteria, we generated candidate compounds using a generative neural network framework^24^. Subsequently, these AI-driven antimicrobials were evaluated using Diptool-based free energy calculations^20^, which evaluated their interactions with two different models of bacterial membrane: one with a randomized distribution of lipids and another with an idealized cardiolipin-rich microdomain.

Our results reveal distinct differences in compound behavior between the two membrane environments. A greater number of compounds exhibited lower binding energy values on microdomain-containing membranes, suggesting that these regions provide favorable binding sites. However, the free energy required for complete translocation was generally higher in the microdomain model, indicating that microdomain-specific properties impede deeper penetration of the compounds. In particular, the cardiolipin-rich domain’s elevated negative charge density and tighter lipid packing strongly attract and hold compounds at the membrane interface via electrostatic and van der Waals forces, yet simultaneously impose a larger energetic barrier to insertion into the hydrophobic core. Indeed, in the CL-rich membrane we observed that compounds with greater polarity (e.g., higher molecular dipole moments) tended to face significantly higher translocation energy barriers, consistent with this anchoring effect, whereas no such correlation was evident in the randomized membrane system. These observations further imply that the localized charge at the hydrophilic headgroup region acts to anchor the molecules firmly at the membrane interface, thereby impeding their deeper translocation into the bilayer interior.

We further refined our analysis through the clustering of compounds based on their free energy profiles, which enabled the identification of groups with the highest antibacterial potential. Detailed structural examination of the most promising clusters revealed recurring motifs (e.g., extended hydrophobic chains and aromatic moieties) that have been linked in the literature with effective antimicrobial activity. Moreover, we evaluated the toxicity profiles of these promising compounds using ProTox 3.0^29^, and several of these candidates fell into the lowest toxicity classes, an essential aspect in the development of new drugs. In parallel, our feature importance analysis provided insight into key physicochemical properties that correlate with drug affinity, particularly emphasizing the role of charge distribution and molecular polarity in strong membrane binding, thereby highlighting structural attributes critical to the potent antimicrobial action.^67^

For additional context, we compared our AI-selected leads with known membrane-targeting antibiotics. We found that the top candidates exhibit membrane interaction profiles analogous to those of established drugs. This parallel suggests that our *de novo* compounds may leverage similar mechanisms as proven antimicrobials, further indicating their therapeutic promise. Equally important, from our screening, we identified three lead molecules that combine potent predicted membrane activity with favorable drug-like properties and low-toxicity predictions. The leading candidates identified in clusters 1 and 2 demonstrate the effectiveness of our design framework and should be prioritized for experimental validation. The absence of experimental confirmation and high-resolution molecular dynamics remains a notable limitation and an essential direction for future work.

Collectively, these findings validate our initial objectives by demonstrating that the integration of generative AI and advanced computational screening can effectively guide the rational design of membrane-targeting antimicrobials. The observed preferential binding of compounds to cardiolipin-rich microdomains, together with the higher energetic cost of membrane penetration in these regions, highlights the important role of membrane composition in drug efficacy. This integrated approach may improve our understanding of structure–activity relationships, particularly how membrane domain composition influences antimicrobial activity and offer a promising framework for the development of new therapeutic agents in the fight against antibiotic-resistant pathogens.

## Supplementary Information

Supplementary Information.

## Data Availability

The full Diptool simulation outputs on domain and non-domain bacterial membranes, the generated compound library, and comprehensive molecular descriptors are publicly accessible in the GitHub repository https://github.com/mrzyckiz/AI-microbials_on_microdomains and are also provided as Supplementary Files.
