# Supplementary material for: Designing AI-generated antimicrobials for targeting bacterial microdomains
Source: Sci Rep. 2025 Dec 9;16:1708. doi: 10.1038/s41598-025-31350-1 (PMC12800277; doi:10.1038/s41598-025-31350-1)
Supplement: Supplementary file 1 — Supplementary Information. [file 41598_2025_31350_MOESM1_ESM.zip › AI_agents_MS_SM.pdf]

Designing AI-Generated Antimicrobials for Targeting Bacterial Microdomains

Mateusz Rzycki[1,*] **and Adam Gruda**[1]

[1]Department of Biomedical Engineering, Wroclaw University of Science and Technology, Wroclaw, 50-370, Poland
[*]mateusz.rzycki@pwr.edu.pl

Lipid dipole moments

Membrane systems were constructed using CHARMM-GUI Membrane Builder[1] to generate an Escherichia coli–like bilayer composed of phosphatidylethanolamine (PYPE), phosphatidylglycerol (PYPG), and cardiolipin (PVCL), following the protocol described in our other work[2]. The assembled bilayer was equilibrated and subjected to 200 ns of all-atom molecular dynamics simulations in GROMACS v.2021[3]. From the equilibrated trajectory, individual lipid molecules were extracted without further geometric optimization to preserve a broad conformational ensemble in dipole calculations[4]. The dipole moments for each lipid conformation were then calculated using the semi-empirical PM7 method implemented in MOPAC[5]. The resulting dipole moment values were averaged and reported as mean $\pm$ standard deviation to provide statistically robust input parameters for Diptool free energy calculations. These mean dipole moments (see Table S1) served for modeling coarse-grained membrane interaction in Diptool.

Table S1. Lipid dipole moments

Lipid type	X dipole [D]	Y dipole [D]	Z dipole [D]
**PYPE**	1.97 $\pm$ 27.36	-1.84 $\pm$ 23.99	-0.39 $\pm$ 10.84
**PYPG**	0.14 $\pm$ 10.17	0.29 $\pm$ 10.09	-35.08 $\pm$ 8.29
**PVCL**	-8.36 $\pm$ 25.39	6.83 $\pm$ 35.44	-28.59 $\pm$ 16.62

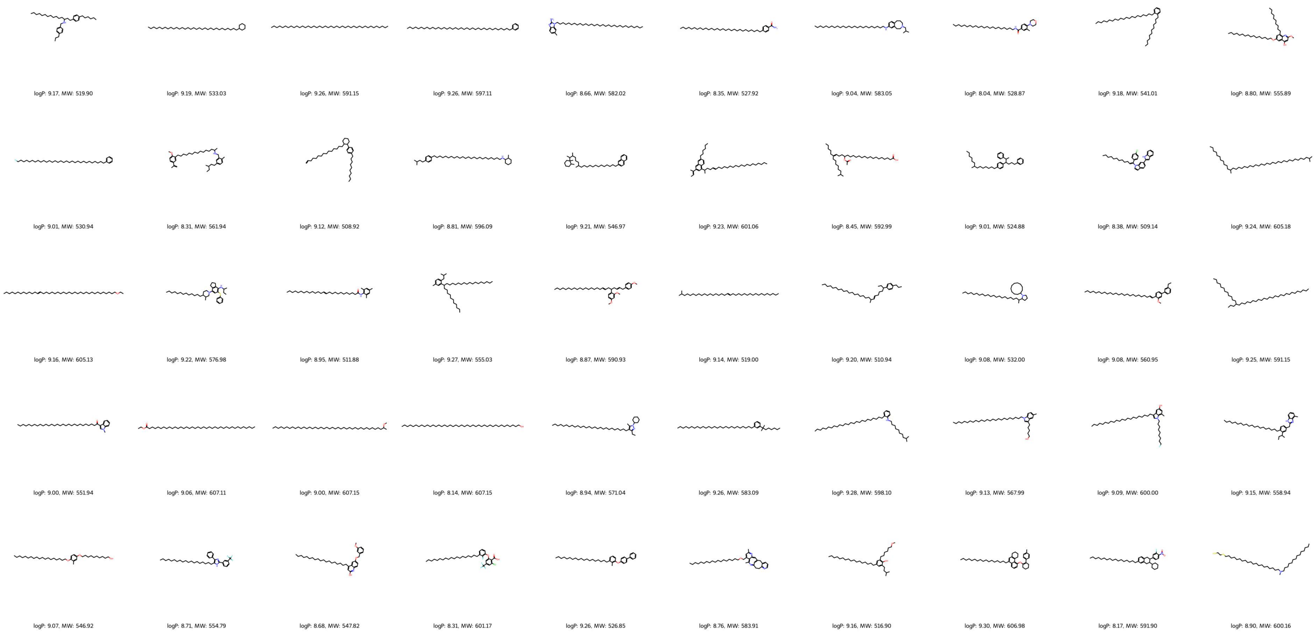

Figure S1. Exemplary AI-generated molecular structures rendered from SMILES strings. These representations of representative compounds were produced by the ReLeaSE generative framework, illustrating key structural motifs.

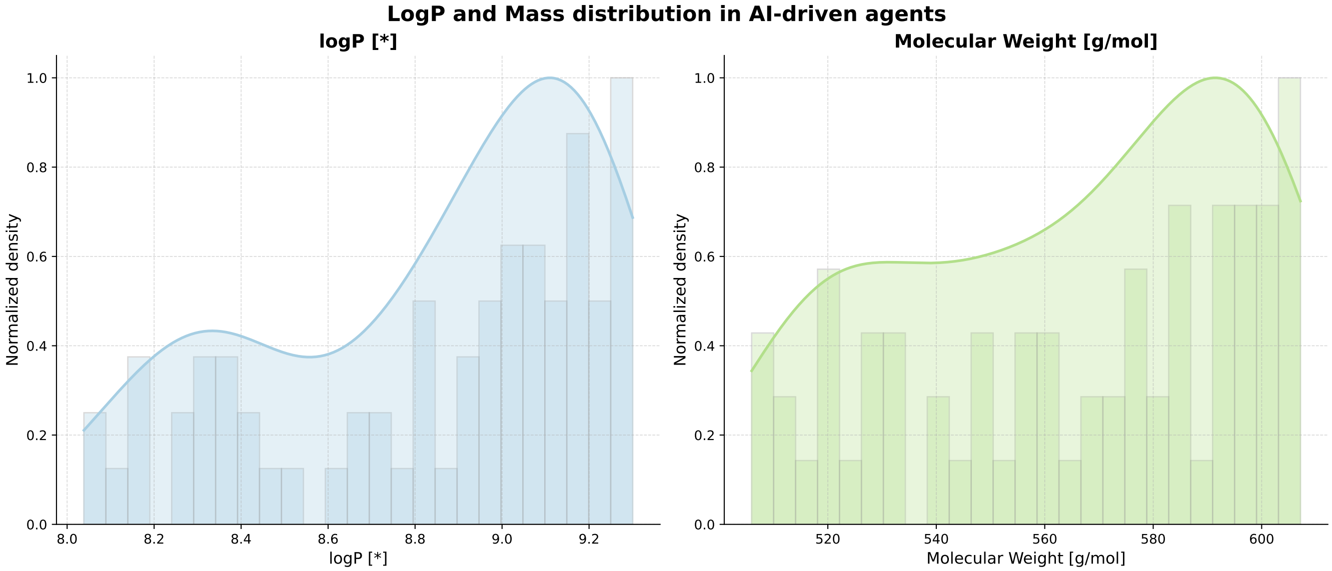

Figure S2. Normalized probability density functions (solid lines) and empirical frequency histograms (bars) of physicochemical properties for the 73 AI-generated candidates. (left) Partition coefficient (log P) distribution, demonstrating enrichment in the design range of 8–11. Shaded regions denote the specified design criteria boundaries. (right) Molecular weight distribution, illustrating that generated compounds cluster within the target 490–610 g/mol window.

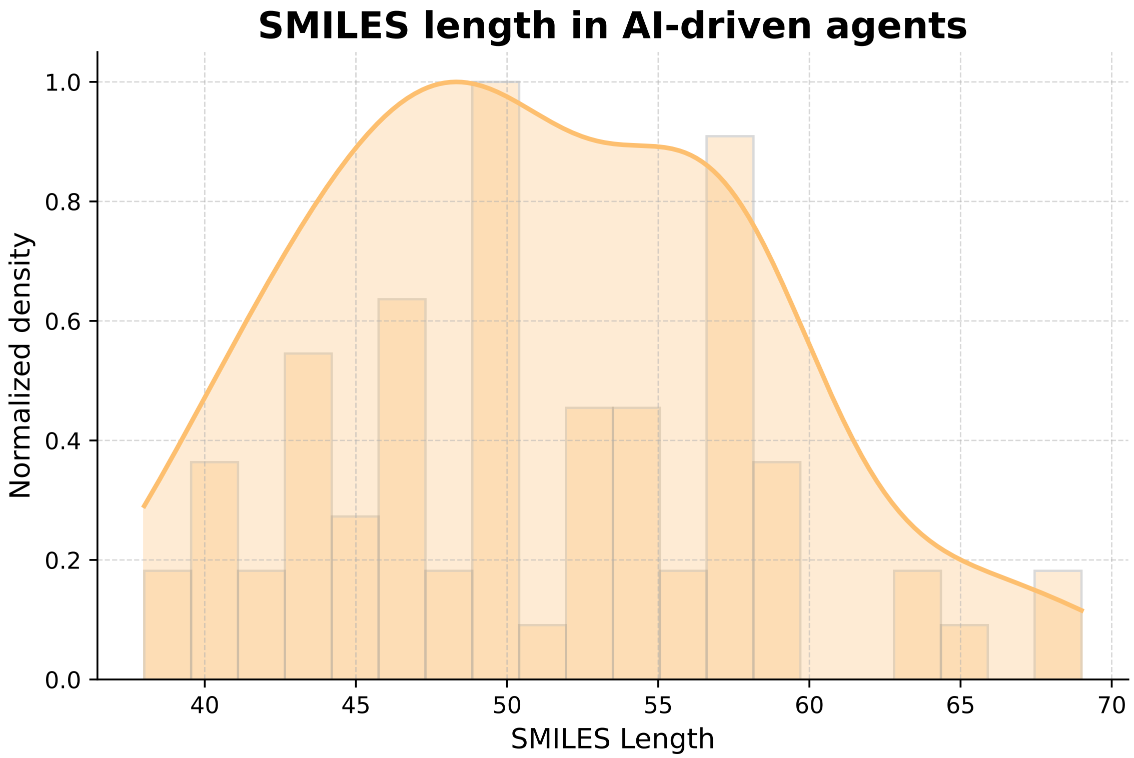

Figure S3. Normalized probability density function (solid line) and empirical frequency histogram (bars) of SMILES string lengths for the 73 AI-generated molecules, given the range and distribution of molecular complexity of agents.

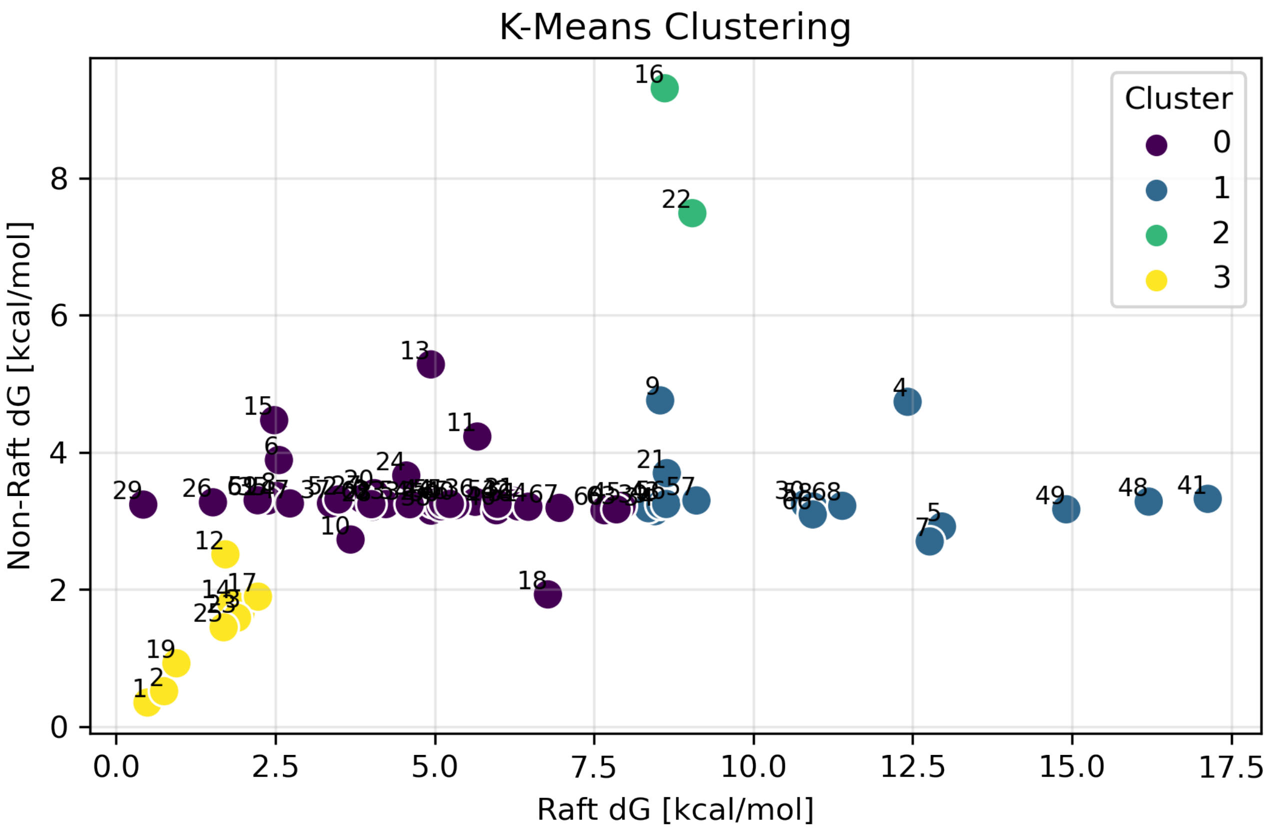

Figure S4. K-means clustering of AI-generated compounds based on full translocation free energies (ΔG_t) through cardiolipin-rich (raft) versus homogeneous (non-raft) membrane models. Each data point corresponds to one of the 73 molecules, plotted by its (ΔG_t) in the raft simulation (x-axis) and in the homogeneous membrane (y-axis). Cluster grouping (0–4) is indicated by color, and numeric labels refer to compound indices as listed in the Supplementary spreadsheet (.csv).

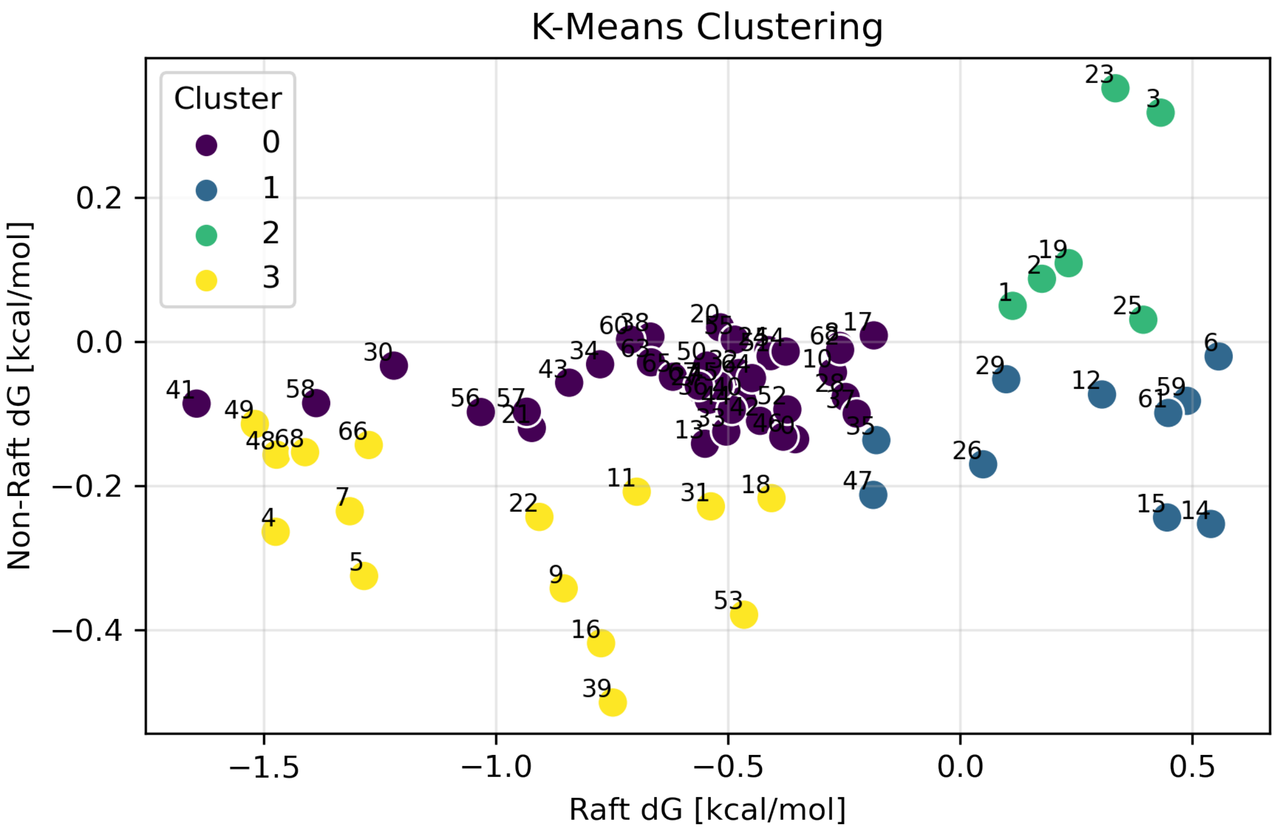

Figure S5. K-means clustering of AI-generated compounds based on minimal approach free energies (ΔG_b) toward cardiolipin-rich (raft) versus homogeneous (non-raft) membranes. Each point represents one molecule, positioned by its (ΔG_b) in the raft model (x-axis) and in the non-raft model (y-axis). Cluster assignments (0–4) are indicated by color, and numeric labels correspond to compound indices in the Supplementary spreadsheet (.csv).

References

1. Wu, E. L. *et al.* CHARMM-GUI <i>Membrane Builder</i> toward realistic biological membrane simulations. *J. Comput. Chem.* **35**, 1997–2004, DOI: 10.1002/jcc.23702 (2014).
2. Rzycki, M., Drabik, D., Szostak-Paluch, K., Hanus-Lorenz, B. & Kraszewski, S. Unraveling the mechanism of octenidine and chlorhexidine on membranes: Does electrostatics matter? *Biophys. J.* **120**, 3392–3408 (2021).
3. Abraham, M. J. *et al.* GROMACS: High performance molecular simulations through multi-level parallelism from laptops to supercomputers. *SoftwareX* **1-2**, 19–25, DOI: 10.1016/J.SOFTX.2015.06.001 (2015).
4. Rzycki, M., Kraszewski, S. & Gładysiewicz-Kudrawiec, M. Diptool—A novel numerical tool for membrane interactions analysis, applying to antimicrobial detergents and drug delivery aids. *Materials* **14**, 6455 (2021).
5. J.P., J. AMS 2024.1 MOPAC: MOPAC Engine based on the MOPAC2016.
